# Supplementary material for: Potential Common Mechanisms of Cytotoxicity Induced by Amide Herbicides via TRPA1 Channel Activation
Source: Int J Environ Res Public Health. 2022 Jun 29;19(13):7985. doi: 10.3390/ijerph19137985 (PMC9266004; doi:10.3390/ijerph19137985)
Supplement: Supplementary file 1 [file ijerph-19-07985-s001.zip › ijerph-1767177-supplementary.pdf]

International Journal of
Environmental Research and Public Health

MDPI

Supplementary Materials

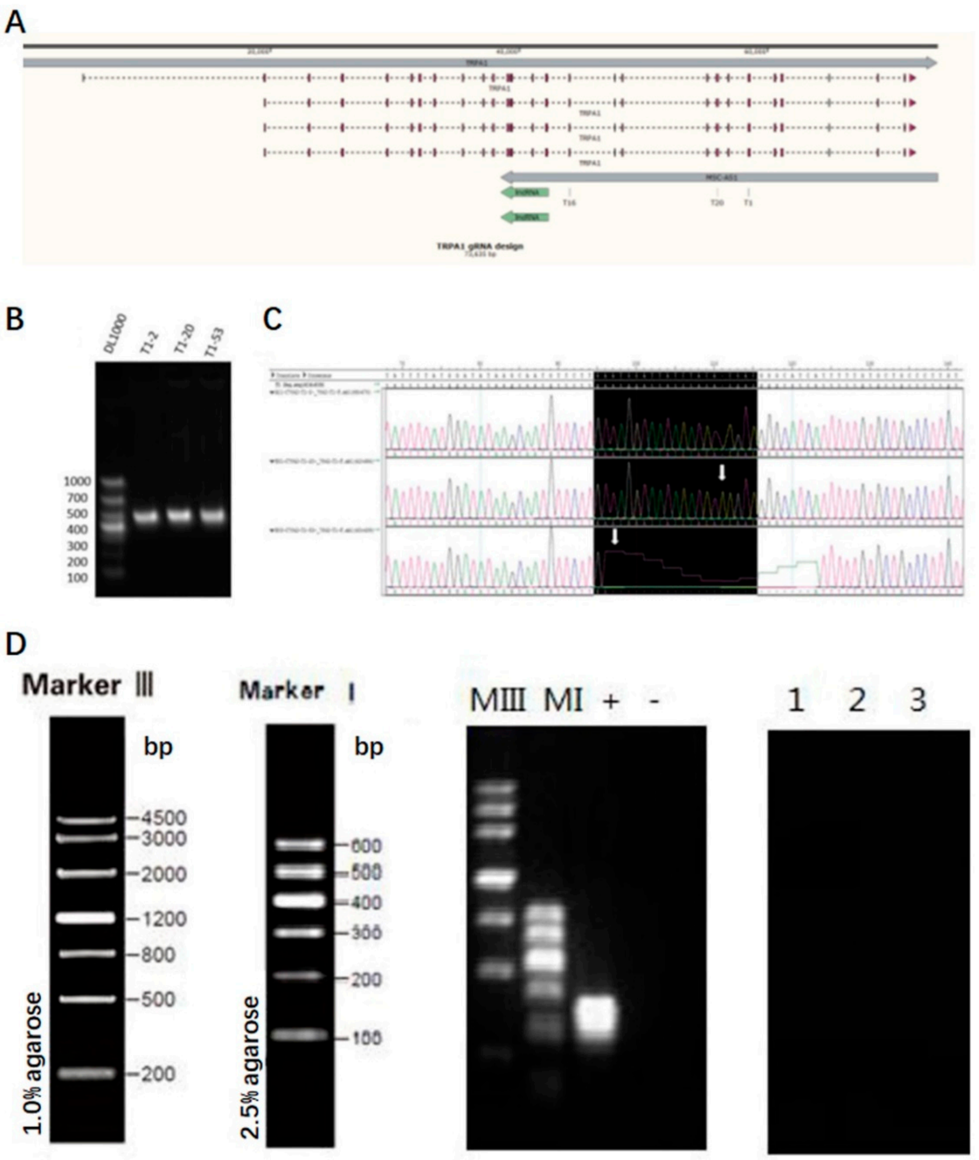

Figure S1. Introduction of construction of TRPA1-KO A549 cells. (**A**) Gene positioning of three gRNAs (T1, T16, T20), arrows indicate direction, genotyping for single cell clone T1-2 (A549 *TRPA1* 0/0), clone T1-20 (A549 *TRPA1* +1/+1), and clone T1-53 (A549 *TRPA1* −25/−25). (**B**) Agarose gel electrophoresis of PCR products. (**C**) Sequence alignment. (**D**) Target location of gRNA is marked by black area. Sites of insertions or deletions (InDel) are marked with white arrows. Agarose gel electrophoresis showing negative mycoplasma test results. Lane +: positive control, Lane −: negative control, Lane 1: TRPA1 knockout positive clone T1-20 (A549 *TRPA1* +1/+1), Lane 2: TRPA1 knockout positive clone T1-53 (A549 *TRPA1* −25/−25); Lane 3: TRPA1 knockout negative clone T1-2 (A549 *TRPA1* 0/0).

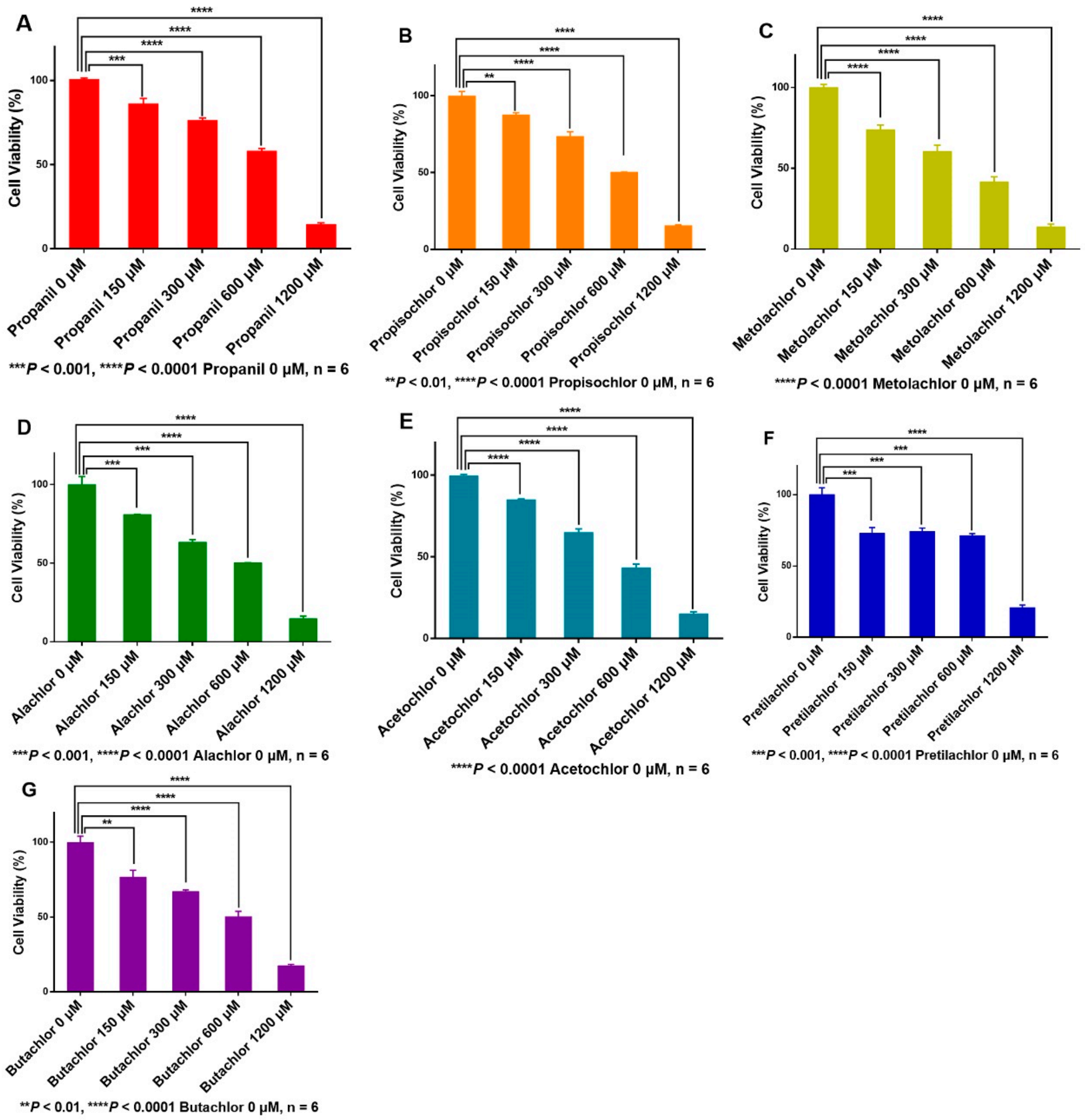

Figure S2. Cell viability of A549 cells treated with AHs. (**A**) Propanil, (**B**) Propisochlor, (**C**) Metolachlor, (**D**) Alachlor, (**E**) Acetochlor, (**F**) Pretilachlor, (**G**) Butachlor.

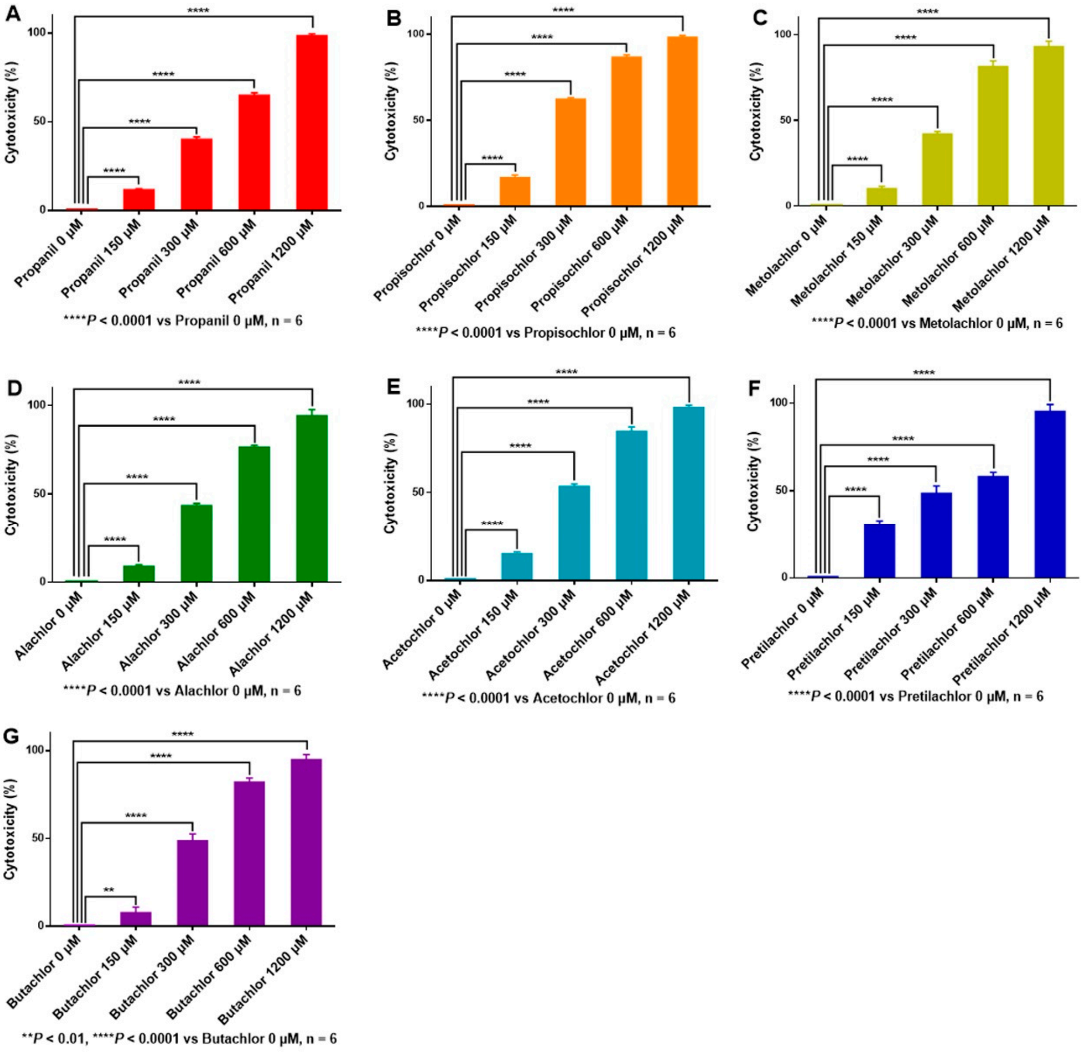

Figure S3. Cytotoxicity of A549 cells treated with AHs. (**A**) Propanil, (**B**) Propisochlor, (**C**) Metolachlor, (**D**) Alachlor, (**E**) Acetochlor, (**F**) Pretilachlor, (**G**) Butachlor.

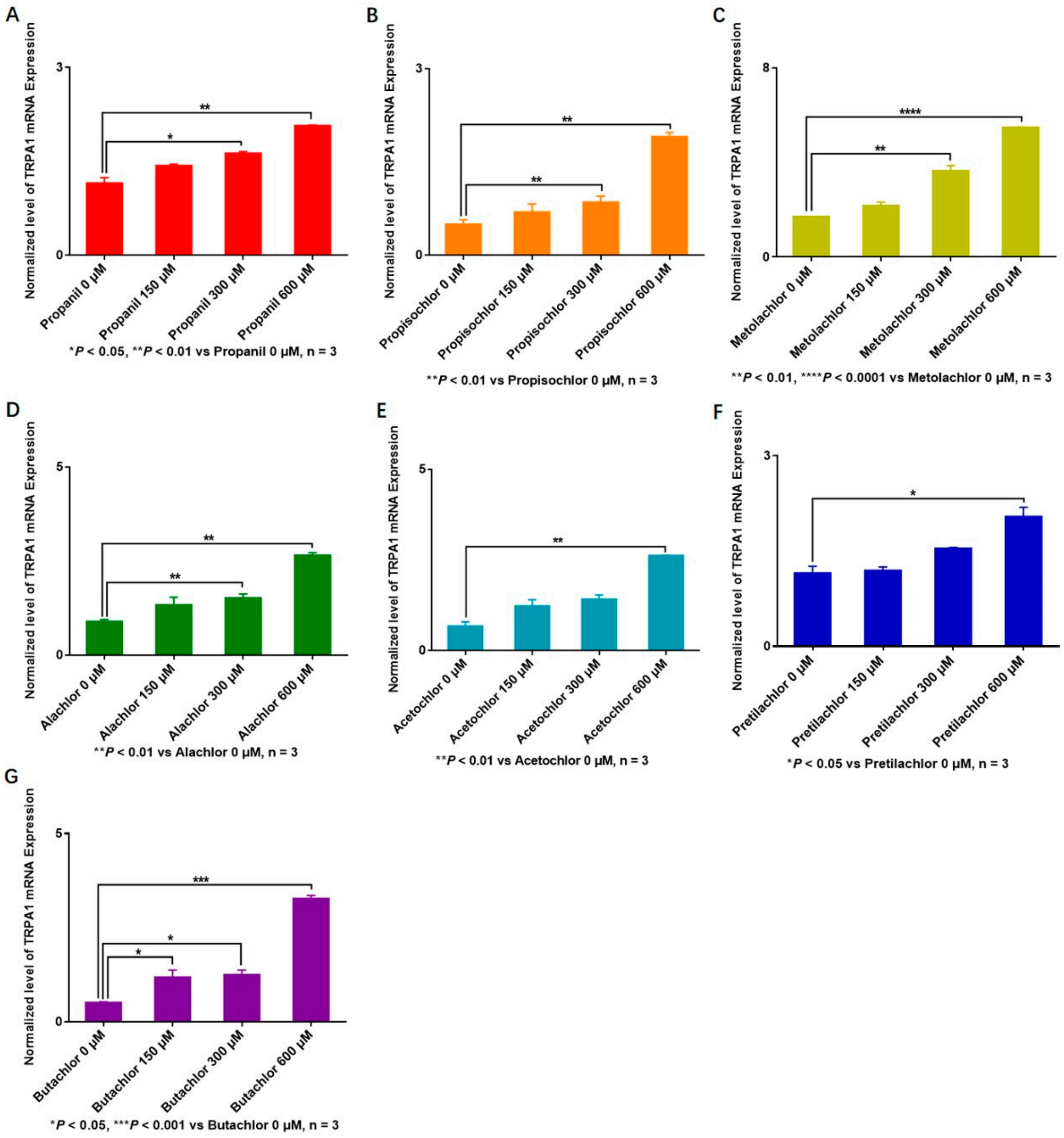

Figure S4. Expression of TRPA1 mRNA in A549 cells treated with AHs. (**A**) Propanil, (**B**) Propisochlor, (**C**) Metolachlor, (**D**) Alachlor, (**E**) Acetochlor, (**F**) Pretilachlor, (**G**) Butachlor.

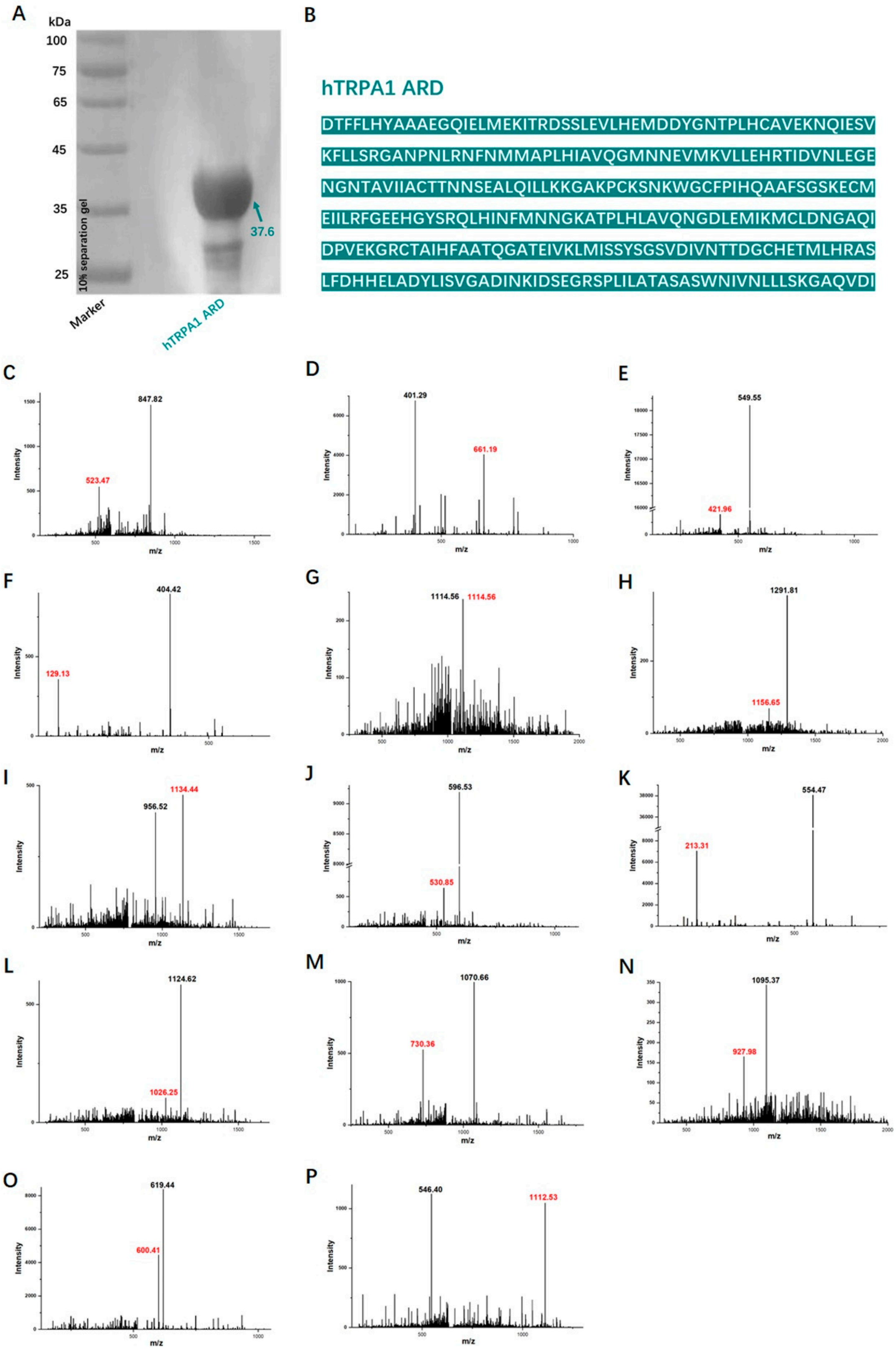

Figure S5. Molecular weight of hTRPA1 ARD as determined by (**A**) SDS-polyacrylamide gel electrophoresis, (**B**) amino acid sequence obtained from NCBI and results of secondary structure for main CID chips and *m*/*z* for hTRPA1 ARD by two-stage mass spectrometry (MS/MS) (**C**–**P**).

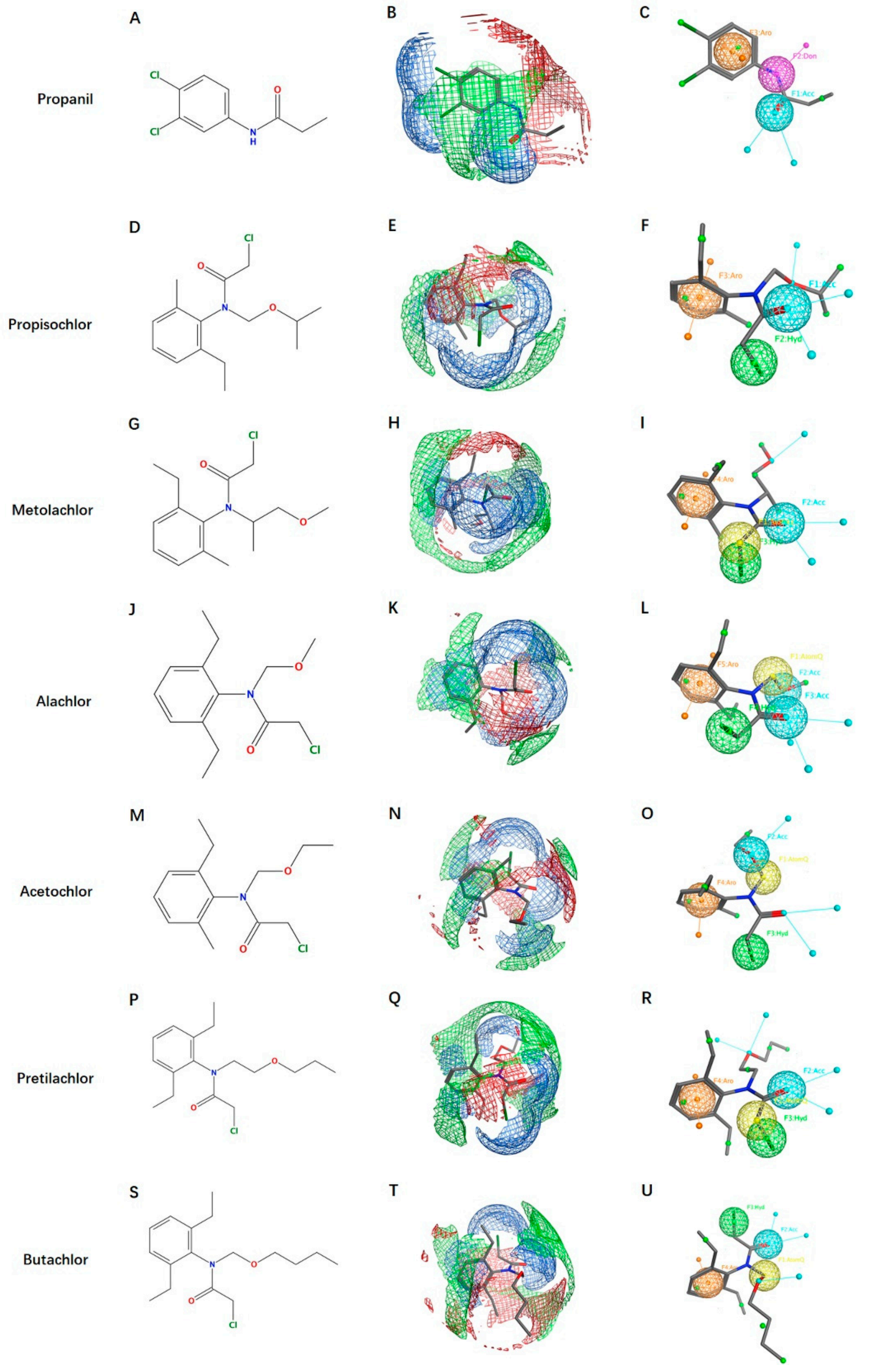

Figure S6. Two-dimensional structure of AHs as Propanil, Propisochlor, Metolachlor, Alachlor, Acetochlor, Pretilachlor, Butachlor ((**A**,**D**,**G**,**J**,**M**,**P**,**S**) respectively). Van der Waals' map of AHs as Propanil, Propisochlor, Metolachlor, Alachlor, Acetochlor, Pretilachlor, Butachlor ((**B**,**E**,**H**,**K**,**N**,**Q**,**T**) respectively), red area represents hydrogen bond and green area represents hydrophobic interaction. Key toxic-effecting groups for AHs interaction with TRPA1 as Propanil, Propisochlor, Metolachlor, Alachlor, Acetochlor, Pretilachlor, Butachlor ((**C**,**F**,**I**,**L**,**O**,**R**,**U**) respectively).

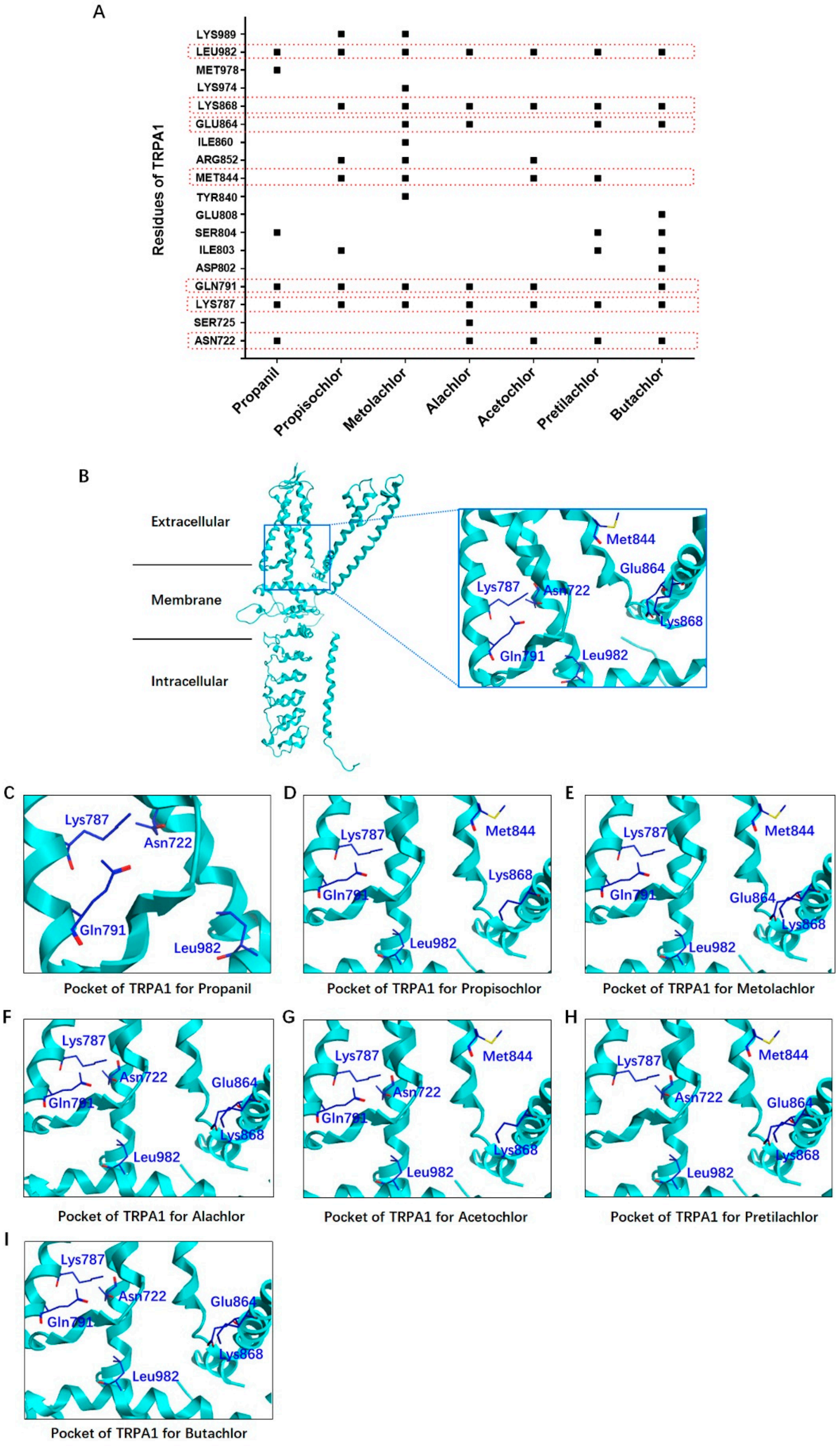

Figure S7. (**A**) Amino acids with high frequency ≥4 for participation in TRPA1-AHs combination. (**B**) Cryo-EM structure of TRPA1 channel. Key interaction pockets of TRPA1 with AHs as (**C**) Propanil, (**D**) Propisochlor, (**E**) Metolachlor, (**F**) Alachlor, (**G**) Acetochlor, (**H**) Pretilachlor, (**I**) Butachlor.

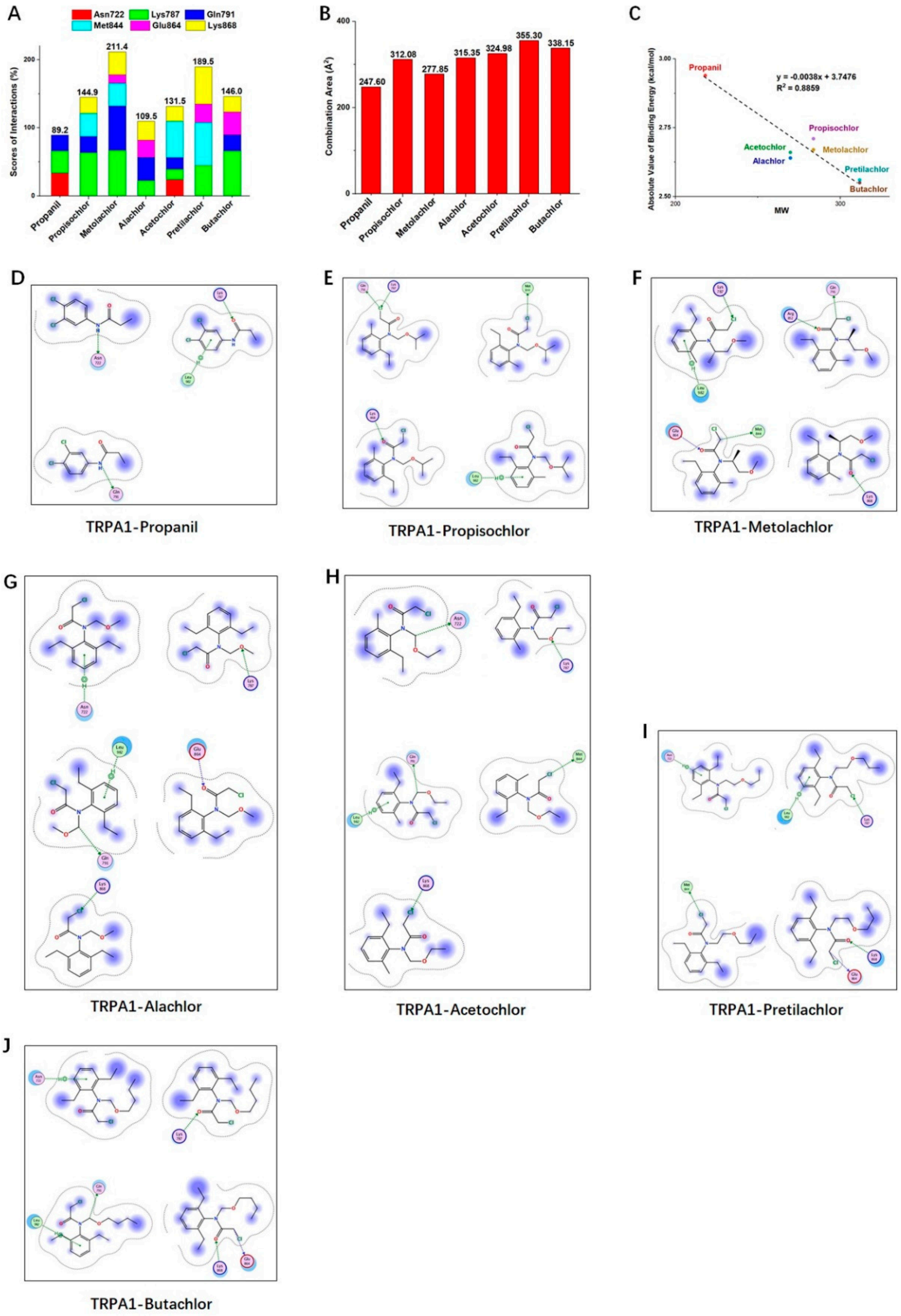

Figure S8. Interactions information between TRPA1 and AHs. (**A**) Hydrogen bonding, (**B**) Combination area, (**C**) Correlation evaluation for TRPA1-AHs by absolute value of binding energy (|BE|) and MW of AHs. Interaction of TRPA1-AHs as two-dimensional structure for (**D**) Propanil, (**E**) Propisochlor, (**F**) Metolachlor, (**G**) Alachlor, (**H**) Acetochlor, (**I**) Pretilachlor, (**J**) Butachlor.

Table S1. Results of secondary structure for main CID chips and *m/z* for TRPA1 ARD detected by MS/MS.

m/z of b^{+}	CID of b^{+}	*m/z* of b^{2+}	CID of b^{2+}	*m/z* of y^{+}	CID of y^{+}	*m/z* of y^{2+}	CID of y^{2+}	*m/z* of y^{3+}	CID of y^{3+}
-	-	523.47	[ATPLHLAVQN+2H]$^{2+}$	-	-	847.82	[PLHLAVQNGDLEMIK+2H]$^{2+}$	-	-
661.19	[ECMEI-H_2O+H]$^{+}$	-	-	401.29	[ILR+H]$^{+}$	-	-	-	-
-	-	421.96	[FLLSRGAN+2H]$^{2+}$	-	-	549.55	[LSRGANPNLR+2H]$^{2+}$	-	-
-	-	129.13	[GAK+2H]$^{2+}$	404.42	[PCK+H]$^{+}$	-	-	-	-
1114.56	[ITRDSSLEVL+H]$^{+}$	-	-	-	-	1114.56	[VLHEMDDYGNTPLHCAVEK+2H]$^{2+}$	-	-
1156.65	[LMISSYSGSVD+H]$^{+}$	-	-	-	-	1291.81	[SSYSGSVDIVNTTDG-CHETMLHR+2H]$^{2+}$	-	-
1134.44	[MCLD-NGAQID+H]$^{+}$	-	-	956.52	[GAQIDPVEK+H]$^{+}$	-	-	-	-
-	-	530.85	[NQIESVKFL+2H]$^{2+}$	-	-	596.53	[IESVKFLLSR+2H]$^{2+}$	-	-
213.31	[VL+H]$^{+}$	-	-	554.47	[LEHR+H]$^{+}$	-	-	-	-
1026.25	[WGCFPIHQ+H]$^{+}$	-	-	1124.62	[PIHQAAFSGSK-H_2O+H]$^{+}$	-	-	-	-
-	-	730.36	[CTAIHFAATQGATE+2H]$^{2+}$	1070.66	[AATQGATEIVK-H_2O+H]$^{+}$	-	-	-	-
-	-	927.98	[DSSLEVLHEMDDYGNT-O+2H]$^{2+}$	-	-	-	-	1095.37	[LEVLHEMDDYGNTPLHCAVEKN-QIESVK+3H]$^{3+}$
600.41	[FGEEH+H]$^{+}$	-	-	619.44	[HGYSR+H]$^{+}$	-	-	-	-
1112.53	[QLHINFMNN+H]$^{+}$	-	-	546.40	[MNNGK-NH_3+H]$^{+}$	-	-	-	-

Table S2. Information regarding interaction between TRPA1 and AHs by computer virtual docking.

Ligand Name	Ligand Atom	Receptor Atom	Receptor Residue	Interaction Type	Score (%)	Distance (Å)	Energy for Specific Amino Acids (kcal/mol)	Binding Area (Å^2)	Binding Energy (kcal/mol)	Molecular Weight
Propanil	N21	OD1	Asn722	H-donor	33.6	3.32	−0.6	247.60	−2.94	218.08
	O20	NZ	Lys787	H-acceptor	32.7	3.10	−6.4			
	N21	OE1	Gln791	H-donor	22.9	3.50	−0.9			
	6-ring	CD2	Leu982	pi-H	-	4.60	−0.5			
Propisochlor	CL3-40	NZ	Lys787	H-acceptor	63.7	3.19	−1.2	312.08	−2.71	283.79
	CL3-40	OE1	Gln791	H-donor	23.5	3.50	−0.8			

	CL3-40	SD	Met844	H-donor	33.9	3.85	−0.5			
	O41	NZ	Lys868	H-acceptor	23.8	3.12	−4.6			
	6-ring	CD2	Leu982	pi-H	-	3.96	−0.5			
Metolachlor	CL3-40	NZ	Lys787	H-acceptor	66.9	3.43	−0.8	277.85	−2.67	283.79
	C37	OE1	Gln791	H-donor	65.3	3.52	−0.5			
	C37	SD	Met844	H-donor	33.2	4.04	−0.7			
	O41	CA	Glu864	H-acceptor	12.8	3.25	−1.4			
	O41	NZ	Lys868	H-acceptor	33.2	3.14	−1.3			
	6-ring	CD2	Leu982	pi-H	-	4.17	−0.5			
Alachlor	6-ring	CB	Asn722	pi-H	-	4.04	−1.0	315.35	−2.64	269.77
	O22-15	NZ	Lys787	H-acceptor	22.8	3.53	−0.5			
	C2-13	OE1	Gln791	H-donor	34.1	3.35	−0.6			
	O38	CA	Glu864	H-acceptor	24.8	3.21	−0.7			
	CL2-36	NZ	Lys868	H-acceptor	27.8	3.14	−1.8			
	6-ring	CD2	Leu982	pi-H	-	4.03	−0.6			
Acetochlor	C1-10	OD1	Asn722	H-donor	23.8	3.35	−0.5	324.98	−2.66	269.77
	O12-12	NZ	Lys787	H-acceptor	15.6	3.27	−1.1			
	C1-10	OE1	Gln791	H-donor	16.9	3.43	−0.5			
	CL3-37	SD	Met844	H-donor	53.2	3.91	−0.6			
	CL3-37	NZ	Lys868	H-acceptor	22.0	3.00	−1.0			
	6-ring	CD2	Leu982	pi-H	-	4.35	−0.6			
Pretilachlor	6-ring	CB	Asn722	pi-H	-	3.89	−0.7	355.30	−2.56	311.85
	CL3-46	NZ	Lys787	H-acceptor	44.8	3.53	−1.3			
	CL3-46	SD	Met844	H-donor	63.1	3.92	−0.6			
	C43	O	Glu864	H-donor	26.8	3.14	−0.6			
	O47	NZ	Lys868	H-acceptor	54.8	2.86	−3.5			
	6-ring	CD2	Leu982	pi-H	-	4.04	−0.5			
Butachlor	6-ring	CB	Asn722	pi-H	-	4.42	−0.5	338.15	−2.55	311.85
	O47	NZ	Lys787	H-acceptor	66.1	3.03	−2.3			
	C1-13	OE1	Gln791	H-donor	23.9	3.27	−0.8			
	C43	O	Glu864	H-donor	33.4	3.16	−1.0			
	O47	NZ	Lys868	H-acceptor	22.6	2.74	−7.0			
	6-ring	CD2	Leu982	pi-H	-	3.62	−0.7			
